# Novel Prognosis and Therapeutic Response Model of Immune-Related lncRNA Pairs in Clear Cell Renal Cell Carcinoma

**DOI:** 10.3390/vaccines10071161

**Published:** 2022-07-21

**Authors:** Gang Wang, Panhong Liu, Jiangfeng Li, Ke Jin, Xiangyi Zheng, Liping Xie

**Affiliations:** 1 Department of Urology, First Affiliated Hospital, School of Medicine, Zhejiang University, Hangzhou 310003, China; lijf@zju.edu.cn (J.L.); 11518360@zju.edu.cn (K.J.); zheng_xy@zju.edu.cn (X.Z.); 2 Department of Cardiology, The Second Affiliated Hospital Zhejiang University School of Medicine, Hangzhou 310009, China; doctorlph@163.com; 3 Cancer Center, Zhejiang University, Hangzhou 310058, China

**Keywords:** immune, ccRCC, lncRNA, prognosis, bioinformatics

## Abstract

Clear cell renal cell carcinoma (ccRCC) is the most common type of renal carcinoma. It is particularly important to accurately judge the prognosis of patients. Since most tumor prediction models depend on the specific expression level of related genes, a better model therefore needs to be constructed. To provide an immune-related lncRNA (irlncRNAs) tumor prognosis model that is independent of the specific gene expression levels, we first downloaded and sorted out the data on ccRCC in the TCGA database and screened irlncRNAs using co-expression analysis and then obtained the differently expressed irlncRNA (DEirlncRNA) pairs by means of univariate analysis. In addition, we modified LASSO penalized regression. Subsequently, the ROC curve was drawn, and we compared the area under the curve, calculated the Akaike information standard value of the 5-year receiver operating characteristic curve, and determined the cut-off point to establish the best model to distinguish the high- or low-disease-risk group of ccRCC. Subsequently, we reassessed the model from the perspectives of survival, clinic-pathological characteristics, tumor-infiltrating immune cells, chemotherapeutics efficacy, and immunosuppressed biomarkers. A total of 17 DEirlncRNAs pairs (AL031710.1|AC104984.5, AC020907.4|AC127-24.4,AC091185.1|AC005104.1, AL513218.1|AC079015.1, AC104564.3|HOXB-AS3, AC003070.1|LINC01355, SEMA6A-AS1|CR936218.1, AL513327.1|AS005785.1, AC084876.1|AC009704.2, IGFL2-AS1|PRDM16-DT, AC011462.4|MMP25-AS1, AL662844.3I|TGB2-AS1, ARHGAP27P1|AC116914.2, AC093788.1|AC007098.1, MCF2L-AS1|AC093001.1, SMIM25|AC008870.2, and AC027796.4|LINC00893) were identified, all of which were included in the Cox regression model. Using the cut-off point, we can better distinguish patients according to different factors, such as survival status, invasive clinic-pathological features, tumor immune infiltration, whether they are sensitive to chemotherapy or not, and expression of immunosuppressive biomarkers. We constructed the irlncRNA model by means of pairing, which can better eliminate the dependence on the expression level of the target genes. In other words, the signature established by pairing irlncRNA regardless of expression levels showed promising clinical prediction value.

## 1. Introduction

Clear cell renal cell carcinoma is one of the most common pathological types of renal cell carcinoma (RCC). Along with the increase in physical examination rate and detection methods, the incidence rate is increasing and its mortality rate is still high [1]. At present, the first line of treatments in a clinical setting is partial or radical nephrectomy for patients with stage I or II ccRCC, and a combination of targeted therapy and/or immunotherapy for those with stage III or IV [2]. It is well-known that gene mutation is one of the initial factors of tumors. In ccRCC, more than 70% of patients were found to have the VHL gene mutation in gene testing [3]. Loss of VHL function can induce hypoxia inducible factors, such as increasing the HIF-1α or HIF-2α increased expression level [4], and through its nuclear transcription factor function, it leads to the abnormal transcription and expression of downstream genes such as VEGF, PDGF, TGF-β, etc. [5]. Therefore, many TKIs and monoclonal antibodies inhibiting VEGFR are used to treat ccRCC patients as first-line drugs, such as sunitinib and sorafenib, etc. [6]. Since a high level of immune cell infiltration was found in ccRCC tissues, the immune sensitivity of ccRCC was further verified by immunological therapy [7]. It is noteworthy that both the INF-α and IL-2 or immune checkpoint inhibitors (ICI) such as PD-1/PD-L1 blockers can significantly improve the overall survival rate (OS) of patients with ccRCC [8]. Recently, the technique of using PD-1/PD-L1 monoclonal antibodies combined with vascular targeted therapy has attracted the attention of many researchers, and it has been demonstrated to be effective in prolonging OS in patients with ccRCC [9]. However, a great deal of research has shown that expression of PD-L1 on the membranes of cancer cells is not an indicator of the clinical outcomes of immunotherapy for ccRCC patients [10], which poses uncertainty regarding when or which immunotherapy drugs should be used. Therefore, it is particularly important to find effective and accurate biological markers to help formulate an individualized treatment schedule.

In recent decades, the emergence of long noncoding RNAs (lncRNAs), a type of RNA strand with nucleotide sequences longer than 200 bases, has received more and more attention [11]. The production method of lncRNAs is similar to that of coding genes and includes a variety of regulatory pathways, such as histone modification and alternative splicing, etc. [12]. Because there is no effective open reading frame, it does not encode any protein [13]. However, this does not affect its biological functions as mRNA or protein expression regulator, the latter including DNA transcription regulation, post transcriptional modification, protein translation, and even participation in epigenetic modification [14]. Therefore, lncRNA is considered to play an indispensable role in the physiology and pathology of organisms, especially in cancer [15]. Recently, increasing evidence has indicated that lncRNAs are involved in the entire progression of ccRCC via a variety of molecular mechanisms, such as alterations of the genomic, transcriptomic, and tumoral immune microenvironment (TIME) [16]. According to reports, the biological markers of a risk model constructed based on the expression of lncRNAs have been demonstrated to predict the OS of patients with cancer, including ccRCC [17].

As immunotherapy, which is a common treatment that is administered in clinical settings to patients with tumors, provides ongoing benefits, signatures that can be used to predict the outcomes of the treatment are receiving increasing focus [18]. For example, signatures such as the infiltration score of immune cells and the expression of immune checkpoints and immune-related genes, etc., have been investigated and reported [19]. In recent studies, immune-related lncRNAs (irlncRNAs) were also considered to establish a signature to predict the prognosis of patients with cancer, for example, hepatocellular cancer [20], gastric cancer [21], pancreatic cancer [22], and even ccRCC [23]. However, these previous characteristics depend on the risk score based on the expression of relevant lncRNAs. Recently, Hong W constructed a novel signature independent of the expression of irlncRNAs in HCC, which was used to predict the prognosis of patients and its correlation with tumor-infiltrating immune cells [24]. However, the application prospects in ccRCC remain to be further studied.

In this study, we utilized a novel modeling algorithm using pairing and iteration to construct an irlncRNA signature that did not require any specific expression levels. Then, we estimated its predictive value among patients with KIRC, as well as its diagnostic effectiveness, chemotherapeutic efficacy, and tumor immune infiltration.

## 2. Result

### 2.1. Screening of Differential Expression of irlncRNA

**Figure 1:**
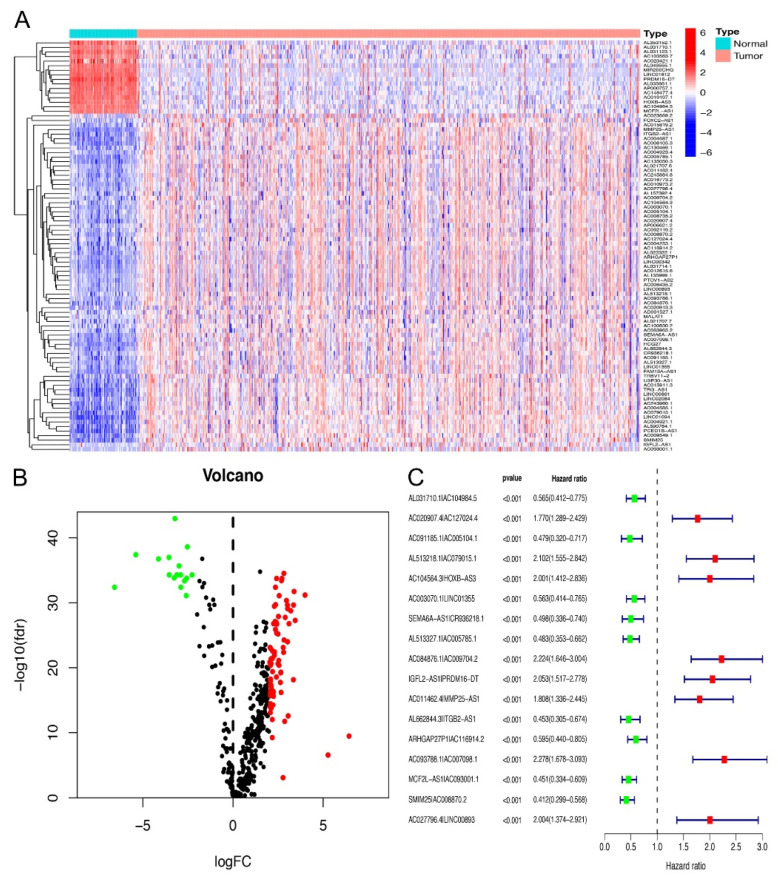
Construction of a risk assessment model using DEirlncRNA pairs. (**A**,**B**) Heatmap (**A**) and Volcano map (**B**) were drawn according to the DEirlncRNA results. (**C**) The Forest map shows 17 DEirlncRNA pairs determined by Cox regression analysis.

We conducted this study in accordance with the following steps. First, we obtained the transcriptome expression profile data of KIRC (as known as ccRCC) from the TCGA database, including 72 normal and 539 tumor samples. Next, we annotated the date according to gene transfer format (GTF) files from Ensembl to convert the gene ID into gene symbols. Soon afterwards, we carried out a co-expression analysis among lncRNAs and known immune-related genes. Finally, we obtained 433 irlncRNAs (correlation coefficient = 0.7, *p* < 0.001, shown in Supplementary Materials S1—irlncRNAs), and 90 were considered as DEirlncRNAs (FDR = 0.001, logFC = 2, Figure 1A), including 74 which were upregulated and 16 which were downregulated (Figure 1B, Supplementary Materials S2—DEirlncRNAs).

### 2.2. Identification of DEirlncRNA Pairs and a Risk Assessment Model

**Figure 2:**
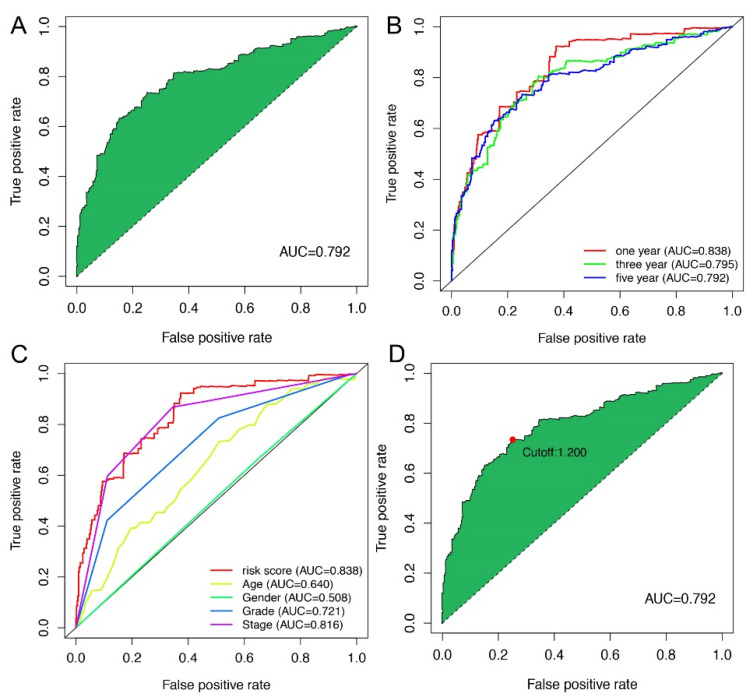
Construction of a risk assessment model using DEirlncRNA pairs. (**A**) The ROC of the optimal DEirlncRNA pair model. (**B**) The 1-, 3-, and 5-year ROC of the optimal model suggested that all AUC values were over 0.792. (**C**) A comparison of 5-year ROC curves with other common clinical characteristics showed the superiority of the riskScore. (**D**) RiskScore for 539 patients with KIRC; the maximum inflection point is the cut-off point obtained by the AIC.

To obtain the DEirlncRNA pairs, we screened the matrix among 90 DEirlncRNA through an iteration loop and a 0-or-1 method. Finally, we obtained 2667 DEirlncRNA pairs (Supplementary Materials S3—DEirlncRNAs pairs). Then, a single-factor analysis was performed, followed by modified LASSO regression analysis; 369 DEirlncRNA pairs were identified, among which 17 were included in the Cox proportional hazard model (Figure 1C). After that, the ROC curve of 17 pairs was drawn, showing that the area under the curve (AUCs) was 0.792 (Figure 2A). To validate our results, we plotted the 1-, 3-, and 5-year ROC curves, respectively, which showed that all of the AUC values were over 0.792 (Figure 2B), and then the 5-year ROC curves were compared with other clinical characteristics (Figure 2C). We recognized the maximum inflection point as the cut-off point on the 5-year ROC curve using the Akaike information criterion (AIC) values (Figure 2D). We collected data of 526 acceptable cases of patients with KIRC from TCGA and calculated the risk scores for all of them. We used the identified cut-off point to re-distinguish high- and low-risk groups in the cohort for validation.

### 2.3. Application of Risk Models in Clinical Evaluation

**Figure 3:**
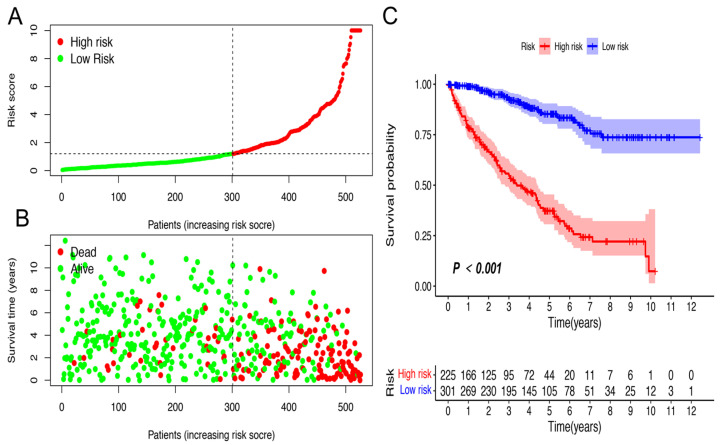
Risk assessment model for predicting prognosis. (**A**) Risk score; (**B**) survival outcome; (**C**) patients. Patients in the low-risk group experienced a longer survival time, as demonstrated by the Kaplan–Meier test.

**Figure 4:**
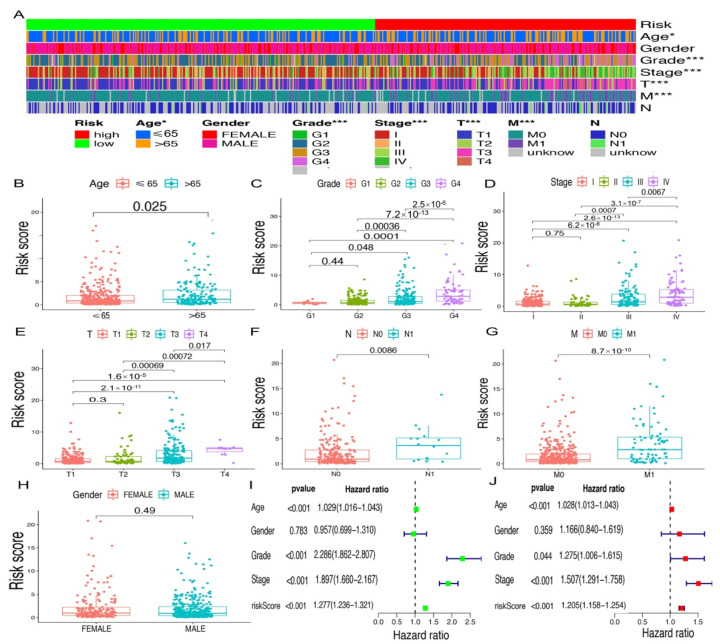
Application of risk assessment model for clinical evaluation. (**A**) Clinical correlation heat map; (**B**) age; (**C**) grade; (**D**) clinical stage; (**E**) T stage; (**F**) N stage; (**G**) M stage; and (**H**) gender. (**I**) The univariate Cox hazard ratio analysis results. (**J**) The multivariate Cox regression analysis results. * *p* < 0.05; *** *p* < 0.001.

On the basis of the above cut-off point, 273 cases were assigned into the low-risk group and 253 cases were assign to the high-risk group. Figure 3A,B show the risk scores and survival of each case. These results suggest that patients in the low-risk group have a better clinical prognosis. Kaplan–Meier analysis further confirmed the above results (*p* < 0.0001) (Figure 3C). Then, we analyzed the relationship between the risk of KIRC and clinicopathological features using a chi-square test. The strip chart (Figure 4A) and consequent scatter diagrams obtained by the Wilcoxon signed-rank test showed that age (Figure 4B), tumor grade (Figure 4C), clinical stage (Figure 4D), T stage (Figure 4E), N stage (Figure 4F), and M stage (Figure 4G) were significantly related to the risk, while gender was not (Figure 4H). Then, through the univariate Cox regression analysis, we found that age (*p* < 0.001, HR = 1.029, 95% CI [1.016–1.043]), clinical grade (*p* < 0.001, HR = 2.286, 95% CI [1.862–2.807]), clinical stage (*p* < 0.001, HR = 1.897, 95% CI [1.660–2.167]), and risk score (*p* < 0.001, HR = 1.270, 95% CI [1.229–1.312]) had significant statistical differences (Figure 4I), whereas in the multivariate Cox regression analysis, risk score (*p* < 0.001, HR = 1.197, 95% CI [1.152–1.245]) was an independent prognostic predictor (Figure 4J). Table S1 shown the specific results of univariate and multivariate Cox regression analyses.

### 2.4. Risk Assessment Model of Tumor-Infiltrating Immune Cells and Immunosuppressive Molecules

**Figure 5:**
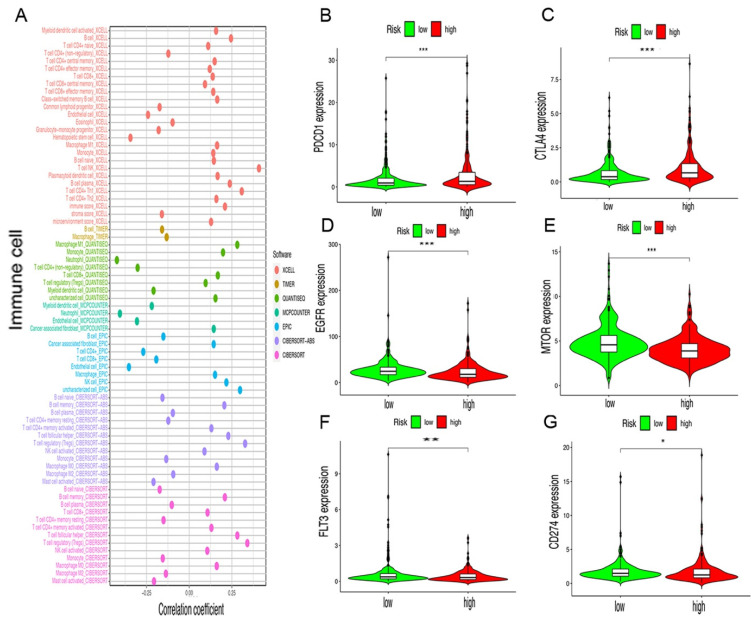
Evaluation of tumor immune cells and immunosuppressive molecules using the risk assessment model. (**A**) Spearman correlation analysis showed the correlation between patients in the high- or low-risk group and immune cells. (**B**,**C**) High risk scores were positively correlated with upregulated (**B**) PDCD1 and (**C**) CD274; (**D**–**F**) high risk scores were negatively correlated with low expression of (**D**) EGFR, (**E**) MTOR, (**F**) FLT3, and (**G**) CD274 levels in patients with KIRC. * *p* < 0.05; ** *p* < 0.01; *** *p* < 0.001.

Since lncRNAs and immune-related genes were initially linked together, we studied whether this model was related to the tumor immune microenvironment. Our study demonstrated that the high-risk groups have a higher correlation with tumor-infiltrating immune cells such as Myeloid dendritic cells, NK cells, CD4^+^ Th1, and T cell NK, whereas they were negatively associated with hematopoietic stem cells, macrophages, and resting memory CD4^+^ cells, as revealed by the Wilcoxon signed-rank test (see Figure S1). Then, we conducted a Spearman correlation analysis, and the resulting diagram shows a lollipop shape (Figure 5A, Table S2). It is well-known that ICIs plays an important role in the treatment of ccRCC; we investigated whether the risk model was related to ICI-related biomarkers and discovered that high risk scores were positively correlated with a high expression of PDCD1 (*p* < 0.001, Figure 5B) and CTLA4 (*p* < 0.001, Figure 5C). However, they were negatively correlated with a low expression of EGFR (*p* < 0.001, Figure 5D), MTOR (*p* < 0.001, Figure 5E), FLT3 (*p* < 0.01, Figure 5F), and CD274 (*p* < 0.05, Figure 5G).

### 2.5. Correlation Analysis between Risk Model and Chemotherapy Drugs

**Figure 6:**
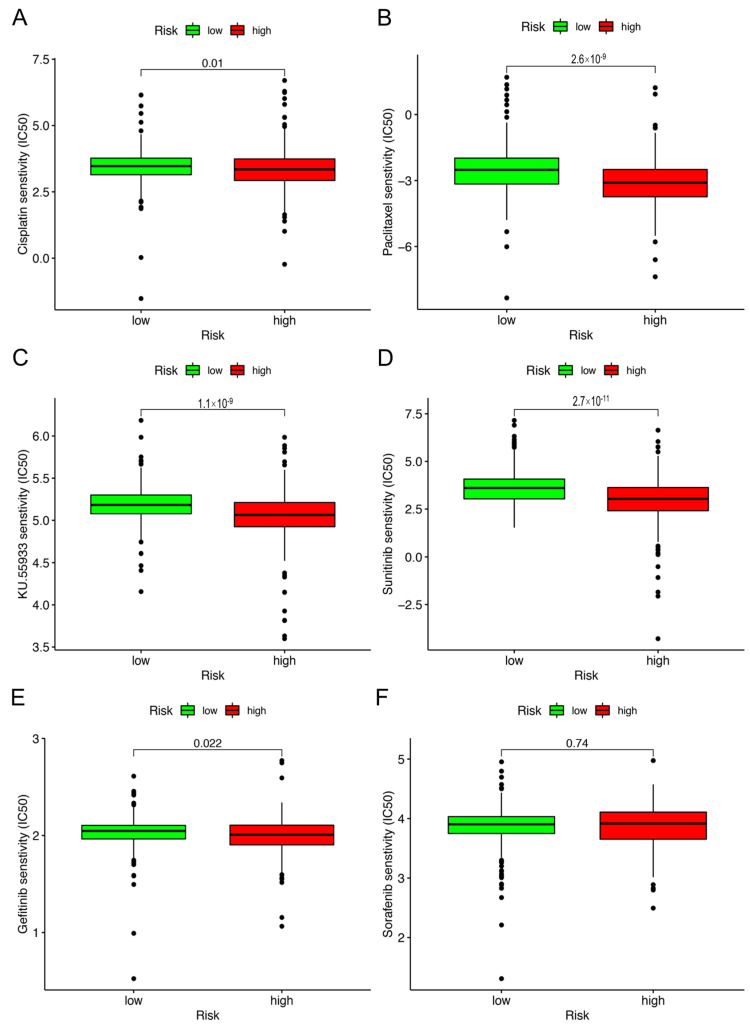
Calculation of the IC50 of KIRC−related drugs using the risk assessment model. The model acted as a potential predictor for chemosensitivity, as high−risk scores were related to a lower IC 50 for chemotherapeutics such as (**A**) cisplatin, (**B**) paclitaxel, (**C**) KU.55933, (**D**) sunitinib, and (**E**) gefitinib. However, (**F**) sorafenib was not statistically significant.

Excluding checkpoint blockade therapy, we sought to determine the association between the risk and efficacy of conventional chemotherapy for KIRC in the TCGA project using the KIRC dataset. We discovered that there is an inverse ratio between the risk score and the half inhibitory concentration (IC50) of chemotherapy agents, for instance, cisplation (*p* = 0.01), paclitaxel (*p* < 0.001), KU.55933 (*p* < 0.001), sunitinib (*p* < 0.001) and gefitinib (*p* = 0.022) (Figure 6A–E), which showed that the model can be used as a potential predictor of chemosensitivity. However, sorafenib (*p* = 0.74) (Figure 6F, Table S3) was not statistically significant.

## 3. Discussion

Along with the rapid development of genomics, an increasing number of genes, including coding genes and lncRNAs, are regarded as novel biomarkers to assist clinicians to formulate therapeutic schedules and evaluate the clinical outcomes of ccRCC patients after different treatments such as surgical operation, targeted therapy, and/or immunological therapy [25]. A recent study suggested that coding genes may be a promising prognostic biomarker of ccRCC because there is a significant positive correlation between their expression and the low survival of ccRCC patients [26]. Meanwhile, other studies pointed out that lncRNAs may act as novel biomarkers for ccRCC diagnosis [27], prognosis [28], and even in targeted therapy [29] and immunological therapy [30]. Previous studies usually explored the TIME and the curative effect of immunotherapy treatment via the signatures established by immune-related genes (irGs) and irlncRNAs. Recently, a study proposed, for the first time, a novel signature based on the irlncRNAs pairs to predict the prognosis of patients with HCC [24], along with lung adenocarcinoma [31,32] and colon adenocarcinoma [33]. We are the first to explore the prospects of this novel model in ccRCC.

In this study, we extracted the expression data of ir-genes and lncRNAs from TCGA database and further obtained 90 DEirlncRNAs, including 74 which were upregulated and 16 which were downregulated, from 433 irLncRNAs by performing a differential co-expression analysis (Figure 2). Then, 17 lncRNA pairs, identified by an iteration loop and the 0-or-1 method from 2667 pairs, were applied to create the ROC curve, and in this way we obtained the most ideal pairs. Third, these results were further validated by the 1-, 3-, and 5-year ROC curves and 5-year clinical ROC curves with clinical characteristics, and then, the best cut-off point was determined according to the maximum AUC value (Figure 3). Finally, we established a risk model according to the cut-off point and divided 526 patients with ccRCC into the high (*n* = 253) and low-risk group (*n* = 273) and analyzed the relationship between the risk score and the overall survival (OS) of patients and clinical pathological characteristics, respectively (Figure 4 and Figure 5). These results revealed that the high-risk subgroups with ccRCC possessed worse OS compared to the low-risk subgroups, as well as higher grades and T- and M-stages, implying the effectiveness of the novel model and the usefulness of the significant irlncRNA pairs as the prognostic biomarker.

Previous research on lncRNAs as biomarkers is mainly divided into two areas. One is concentrated in a single abnormally expressed lncRNA, considering that it may potentially participate in the molecular mechanism of renal clear cell carcinogenesis and development. For example, Hong et al. considered that lncRNA HOTAIR may pass through the mir-217/HIF-1α/Axl signaling pathway, promoting the occurrence of ccRCC [34]. Hirata et al. considered that lncRNA MALAT1 promotes the invasiveness of ccRCC through the miR-205/EZH2 axis [35]. Dong et al. pointed out that lncRNA GAS5 may act as a competitive endogenous RNA (ceRNA) in competitive binding with miR-223 and indirectly regulate hZIP1, so as to regulate the progression of ccRCC [36]. Gang Wang et al. demonstrated that lncRNA OTUD6B-AS1 not only indicates poor prognosis but also inhibits ccRCC proliferation via the Wnt/β-catenin signaling pathway [37]. The other category pays more attention to multiple abnormally expressed lncRNAs, believing that their combination may better improve the predictive value of OS in ccRCC. For instance, Zeng et al. exploited a risk model according to the expression of six novel lncRNAs (CTD-2263F21.1, LINC01510, CTA-384D8.35, RP11-395B7.2, RP11-352G9.1, and RP11-426C22.4), which showed advantageous prognostic value for ccRCC [38]. Qu et al. constructed a classifier based on the expression of four lncRNAs (ENSG00000255774, ENSG00000248323, ENSG00000260911, and ENSG00000231666), which has magnificent potential for predicting the OS of patients with stage I-III ccRCC [39]. However, as previously mentioned, the efficiency of these markers based on lncRNA is mainly affected by their own expression levels. To circumvent this limitation, we performed a novel risk model dependent on the 17 irlncRNAs pairs obtained through the iteration loop, 0-or-1 method, and LASSO regression analysis, of which the critical value was acquired according to the cut-off point and implemented to divide patients into different risk groups. This novel model was not only independent of the expression level of irlncRNAs, but also did not distinguish risk groups just by the median value of the risk score. Additionally, we found that the model had the advantage of clinical applicability by analyzing the relationship between risk subgroups and clinicopathological characteristics in ccRCC.

Some studies have shown the potential function of lncRNAs to regulate the TIME in ccRCC [40]. For instance, lncRNA MIR155HG is thought to be associated with immune checkpoint expression and immune cell infiltration in ccRCC [41]. Furthermore, an increasing number of irlncRNAs are used in the promising and important prognosis prediction of ccRCC patients, including the potential clinical value of immunotherapy [42,43]. For instance, Zhong et al. suggested that 14 irlncRNAs are a prognostic signature to evaluate the OS of ccRCC patients because of their relationship to the level of immune cell infiltration [44]. Therefore, considering that the targeted genes in this study were the irlncRNAs pairs, we consequently investigated the features of TIME in high/risk subgroups with ccRCC produced based on the risk model. First, we estimated the immune cell infiltration in high-risk subgroups using seven pieces of software and found that most types of immune cells prefer to infiltrate in subgroups with high risk (Figure 6) (which was consistent with previous research results [45]), indicating that more numerous immune cells infiltrate into the tumor tissue of patients with more advanced ccRCC whether they are immune killer cells (such as CD8^+^ T cell) or immunosuppressive cells (such as regulatory T cell). Meanwhile, we discovered that the classical immune checkpoint genes including PD-1 (PDCD1) and PD-L1 (CD274) were present at a higher level in subgroups with high risk, which hinted that immune cells infiltrating into tumor tissue may lose their immunogenicity, resulting in the appearance of immunosuppression. As Braun DA argued, not all immune killer cells infiltrating into ccRCC can fulfill effective lethality [46]. In addition, the model also suggested that subgroups with high risk were non-sensitive to chemotherapeutics and targeted therapeutics, which reflected the characteristics of ccRCC patients with regard to drugs, consistent with this phenomenon in clinical settings.

In addition, our research still has a few limitations, similar to the previous studies. First of all, the deviation of the consequences can not be ignored because all of the data were obtained from public resources using the public R package. Second, there are currently no ccRCC samples for the treatment of immunotherapy drugs in the TCGA database, so the novel model can not reflect the sensitivity of ccRCC patients to immunotherapy drugs. Finally, the 14 significant irlncRNAs pairs based on the risk model lacked clinical data validation due to all of the data being obtained from public databases; this will be solved in the future by recollecting ccRCC samples in clinical settings and further verifying the validity of these signatures.

In conclusion, this study demonstrated that a novel signature constructed in irlncRNAs and that does not require the prediction of lncRNA expression levels could predict prognosis for patients with KIRC and might help in distinguishing those who could benefit from anti-tumor immunotherapy.

## 4. Materials and Methods

### 4.1. Obtained, Sorted, and Differential Expression Analysis of Transcriptome Data

First, we obtained the KIRC transcriptome data from the TCGA database, which is consistent with fragment perkilobase million (FPKM). Next, for follow-up analysis, we gained the GTF files from the Ensemble database to distinguish between lncRNA and mRNA. After that, the known immune-related genes (ir-genes) were downloaded from ImmPort database (http://www.immport.org, accessed on 14 Febrary 2022) to facilitate the screening of immune-related lncRNA through co-expression methods. Finally, we performed a co-expression analysis between all lncRNA and ir-genes. The lncRNAas with a correlation coefficient greater than 0.7 and a *p* value less than 0.001 were assigned as irlncRNAs. We used R-package limma to analyze the differential expression of irlncRNAs to obtain DEirlncRNA. The thresholds were set to log fold change (FC) >2 and the false discovery rate (FDR) <0.001.

### 4.2. Construction of DEirlncRNAs Pairs

The DEirlncRNAs were cyclically singly paired. In short, we divided the expression of lncRNA-A by lncRNA-B to obtain the coefficient C. If C was greater than 1, it was marked as 1, and otherwise it was 0, and so we could obtain a 0-or-1 matrix. Then, we further analyzed the matrix. No relationship was considered between pairs and prognosis if the expression quantity of lncRNA pairs was 0 or 1 because pairs without a certain rank could not properly predict patient survival outcome. It was considered a valid match if the amount of lncRNA pairs with 0 or 1 expression represented more than 20% of all the pairs.

### 4.3. Acquisition of Patients’ Clinical Data

We downloaded the clinical data of KIRC patients from the TCGA database. The valid data were obtained by deleting data with a follow-up time of 0 days, as well as duplicates.

### 4.4. Construct of a Risk Model for Assessment of the Risk Score

First, univariate analysis was performed, followed by 10-fold cross-validation of LASSO regression, with a *p* value of 0.05. LASSO regression was performed for 1000 cycles, and 1000 times random stimulations were set in each cycle. Next, we recorded the frequency of each pair in the 1000-times-repeated LASSO regression model, and pairs with a frequency of more than 100 times were selected for Cox proportional hazards regression analysis, and then we built the model. Then, we drew the ROC curve of each model and calculated its AUC value. If the curve reached the highest point, meaning the maximum AUC value, the calculation process was terminated, and the model was taken as the best candidate model. The ROC curves of 1-, 3-, and 5-years were drawn. We calculated the risk score of the risk model for all clinical cases by the following formula: RiskScore = $\;\overset{k}{\underset{i=1}{\sum }}\beta iSi$. In order to determine the maximum inflection, the AIC values of every point of the 5-year ROC curve were evaluated, and these were defined as the cut-off point to distinguish between high or low risk scores.

## 5. Validation of the Risk Model

To verify the cut-off point, Kaplan–Meier analysis was performed to show the difference between patients in the high- or low-risk group, as well as visualization of the survival curve. The R tool was used to visualize the specific risk score values of each sample in the model. The R packages applied in these procedures contain survminer, survival, survivalROC, pbapply, glmnet, and pheatmap.

Next, we analyzed the relationship between the model and clinicopathological characteristics by means of a chi-square test to better verify the availability of our model. The bar chart was used for visualization and was marked as follows: <0.001 = ***, <0.01 = **, and <0.05 = *. The risk score differences among different clinicopathological characteristics were computed by the Wilcoxon signed-rank test. The results were shown with a box diagram. Furthermore, we performed univariate and multivariate analysis among the clinicopathological features and risk score, which confirmed that the model can be used as an independent predictor of clinical prognosis, and the result was shown in a forest map. The R packages used in these analyses were survival, pHeat-map, and ggupbr.

### 5.1. Studies on Tumor-Infiltrating Immune Cells

It is well-known that tumor immunization plays a significant role in the development of tumors. In order to apply our risk model to tumor immune cells, we took currently accepted algorithms into account to calculate the immune infiltration statuses among the samples from the TCGA project of the KIRC dataset, including TIMER, CIBERSORT, XCELL, QUANTISEQ, MCPcounter, EPIC, and CIBERSORT. The difference in the content of immune-infiltrating cells between high- and low-risk groups was analyzed by Wilcoxon signed-rank test; The results are shown in a box chart. The correlation between risk score and immune infiltrating cells was analyzed by Spearman correlation analysis. The results of the correlation coefficient were visualized by using a LollipopT chart, where *p* < 0.05 was considered statistically significant. The R package used in these analyses was ggplot2.

### 5.2. Guiding Significance of the Model for Clinical Treatment

To assess the clinical value of the model in the treatment of ccRCC, we calculated the IC50 of commonly administrated chemotherapeutic drugs in the TCGA project based on the KIRC dataset. Antitumor drugs such as cisplatin, paclitaxel, KU.55933, sunitinib, gefitinib and sorafenib are recommended for liver cancer treatment by the AJCC guidelines. The difference in IC50 between high- and low-risk groups was compared by the Wilcoxon signed-rank test; the results are shown as box charts. The R packages used in these analyses were pRRophetic and ggplot2.

## Data Availability

The original contributions presented in the study are included in the article/Supplementary Material. Further inquiries can be directed to the corresponding author (wanggang1990@zju.edu.cn).

## Supplementary Materials

The following supporting information can be downloaded at: https://www.mdpi.com/article/10.3390/vaccines10071161/s1, Figure S1: The representative results of the evaluation of tumor infiltrating immune cells with risk assessment model; Table S1: Univariable and multivariable Cox regression analyses in KIRC; Table S2: The detail comparison results of correlation ship between tumor infiltrating immune cells and risk sore.
