# Supplementary material for: Novel Prognosis and Therapeutic Response Model of Immune-Related lncRNA Pairs in Clear Cell Renal Cell Carcinoma
Source: Vaccines (Basel). 2022 Jul 21;10(7):1161. doi: 10.3390/vaccines10071161 (PMC9325030; doi:10.3390/vaccines10071161)
Supplement: Supplementary file 1 [file vaccines-10-01161-s001.zip › Supplemental Information.pdf]

Supplemental Information:

Novel Prognosis and Therapeutic response model of Immune-related lncRNA pairs in Clear Cell Renal Cell Carcinoma

Gang Wang[1], Panhong Liu[2], Jiangfeng Li[1], Ke Jin[1], Xiangyi Zheng[1], Liping Xie[1]

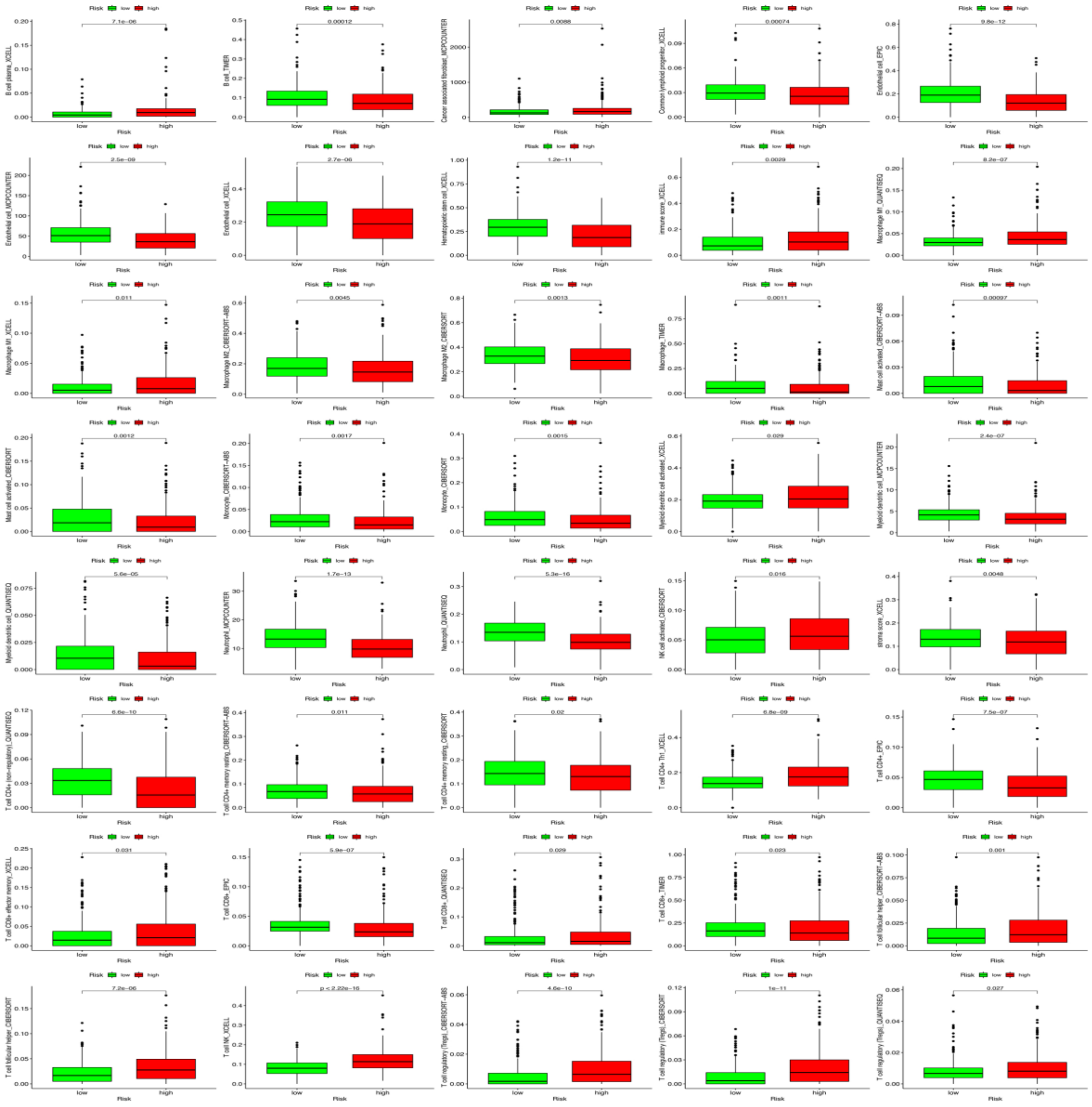

Figure S1. The representative results of the evaluation of tumor infiltrating immune cells with risk assessment model.

Table S1: Univariable and multivariable Cox regression analyses in KIRC.

Characteristics	Univariate analysis			Multivariate analysis		
	HR	95% CI	pvalue	HR	95% CI	pvalue
Age	1.02926	1.01582-1.04287	0.00002	1.02803	1.01312-1.04316	0.00021
Gender	0.95679	0.69889-1.30986	0.78284	1.16609	0.83965-1.61943	0.35916
Grade	2.28613	1.86217-2.80660	0.00000	1.27487	1.00643-1.61489	0.04410
Stage	1.89681	1.65995-2.16747	0.00000	1.50680	1.29129-1.75829	0.00000
riskScore	1.27745	1.23578-1.32052	0.00000	1.20467	1.15751-1.25376	0.00000

In both univariable and multivariable Cox regression analyses, age, gender, grade, stage and riskScore, Expression were evaluated as continuous variables. $P < 0.05$ was considered statistically significant in all analyses.

Table S2: The detail comparison results of correlation ship between tumour infiltrating immune cells and risk sore.

Immune cells	Type	Correlation	p-value
B cell	TIMER	-0.1589881	0.00025125
Macrophage	TIMER	-0.1321237	0.00239478
B cell naive	CIBERSORT	-0.1729378	6.69E-05
B cell memory	CIBERSORT	0.20900677	1.33E-06

B cell plasma	CIBERSORT	-0.1018875	0.01942441
T cell CD8+	CIBERSORT	0.10741859	0.01370566
T cell CD4+ memory resting	CIBERSORT	-0.1501841	0.00054856
T cell CD4+ memory activated	CIBERSORT	0.1301107	0.00279281
T cell follicular helper	CIBERSORT	0.28179604	4.66E-11
T cell regulatory (Tregs)	CIBERSORT	0.34056042	9.48E-16
NK cell activated	CIBERSORT	0.10593058	0.01507605
Monocyte	CIBERSORT	-0.1548498	0.00036454
Macrophage M0	CIBERSORT	0.16128406	0.00020357
Macrophage M2	CIBERSORT	-0.1365467	0.00169572
Mast cell activated	CIBERSORT	-0.206036	1.89E-06
B cell naive	CIBERSORT-ABS	-0.1566614	0.00031008
B cell memory	CIBERSORT-ABS	0.20579494	1.94E-06
B cell plasma	CIBERSORT-ABS	-0.0957489	0.02810671
T cell CD4+ memory resting	CIBERSORT-ABS	-0.1222734	0.00498233
T cell CD4+ memory activated	CIBERSORT-ABS	0.13002696	0.00281061
T cell follicular helper	CIBERSORT-ABS	0.22890281	1.11E-07
T cell regulatory (Tregs)	CIBERSORT-ABS	0.32749205	1.29E-14
NK cell activated	CIBERSORT-ABS	0.08937723	0.04045578

Monocyte	CIBERSORT-ABS	-0.1343951	0.0020083
Macrophage M0	CIBERSORT-ABS	0.16169439	0.000196
Macrophage M2	CIBERSORT-ABS	-0.0933823	0.03225153
Mast cell activated	CIBERSORT-ABS	-0.2089517	1.34E-06
Macrophage M1	QUANTISEQ	0.28154893	4.85E-11
Monocyte	QUANTISEQ	0.19805701	4.72E-06
Neutrophil	QUANTISEQ	-0.4234731	2.65E-24
T cell CD4+ (non-regulatory)	QUANTISEQ	-0.3019058	1.51E-12
T cell CD8+	QUANTISEQ	0.16776862	0.00011063
T cell regulatory (Tregs)	QUANTISEQ	0.09631144	0.0271917
Myeloid dendritic cell	QUANTISEQ	-0.2086886	1.38E-06
uncharacterized cell	QUANTISEQ	0.15391113	0.00039615
Myeloid dendritic cell	MCPCOUNTER	-0.2191264	3.87E-07
Neutrophil	MCPCOUNTER	-0.405708	2.93E-22
Endothelial cell	MCPCOUNTER	-0.305794	7.55E-13
Cancer associated fibroblast	MCPCOUNTER	0.14393556	0.00093123
Myeloid dendritic cell activated	XCELL	0.15716339	0.0002964
B cell	XCELL	0.24469266	1.31E-08
T cell CD4+ naive	XCELL	0.11107104	0.01079668

T cell CD4+ (non-regulatory)	XCELL	-0.1219773	0.00508935
T cell CD4+ central memory	XCELL	0.14904808	0.00060489
T cell CD4+ effector memory	XCELL	0.12060827	0.00561167
T cell CD8+	XCELL	0.13751102	0.00157067
T cell CD8+ central memory	XCELL	0.09230036	0.03431391
T cell CD8+ effector memory	XCELL	0.14098965	0.00118665
Class-switched memory B cell	XCELL	0.16428679	0.00015393
Common lymphoid progenitor	XCELL	-0.1733453	6.43E-05
Endothelial cell	XCELL	-0.2411996	2.12E-08
Eosinophil	XCELL	-0.0972864	0.02566657
Granulocyte-monocyte progenitor	XCELL	-0.1789821	3.65E-05
Hematopoietic stem cell	XCELL	-0.34388	4.78E-16
Macrophage M1	XCELL	0.16335175	0.00016801
Monocyte	XCELL	0.14106623	0.00117926
B cell naive	XCELL	0.14457396	0.00088305
T cell NK	XCELL	0.40857774	1.39E-22
Plasmacytoid dendritic cell	XCELL	0.16634154	0.00012677
B cell plasma	XCELL	0.23760305	3.48E-08
T cell CD4+ Th1	XCELL	0.3077537	5.30E-13

T cell CD4+ Th2	XCELL	0.15915894	0.00024737
immune score	XCELL	0.20962918	1.23E-06
stroma score	XCELL	-0.1600294	0.00022846
microenvironment score	XCELL	0.12805948	0.00325953
B cell	EPIC	-0.1511902	0.00050278
Cancer associated fibroblast	EPIC	0.14355903	0.00096078
T cell CD4+	EPIC	-0.2689344	3.63E-10
T cell CD8+	EPIC	-0.1924836	8.76E-06
Endothelial cell	EPIC	-0.3531751	6.75E-17
Macrophage	EPIC	0.15131515	0.00049735
NK cell	EPIC	0.21773541	4.60E-07

Table S3. The p value of comparing chemotherapeutics sensitivity and risk sore.

Drug	p-value
Cisplatin	0.01
Gefitinib	0.022
KU.55933	1.1e-9
Paclitaxel	2.6e-9
Sorafenib	0.74

Sunitinib	2.7e-11
